# Notes on Parasites

**Published:** 1892-08

**Authors:** C. W. Stiles

**Affiliations:** Bureau of Animal Industry, U. S. Department of Agriculture


### NOTES ON PARASITES.

C. W. Stiles, Ph. D.

No. 11. Distoma magnum Bassi, 1875.

In the March number of this Journal, and in the *American Veterinary Review* of the same date, I stated that Dr. Francis's *Distomum Texanicum* (October 1891) was the same parasite Dr. Hassall described in July, 1891, as *Fasciola carnosa*, and of which he afterwards changed the name to *F. Americana* ; in the same note I expressed the opinion that this American fluke was identical with *D. magnum*.

Since the publication of that note, Prof. Sonsino, the learned helminthologist of Pisa, Italy, has had the extreme kindness to send me eight specimens of *D. magnum* which both Dr. Hassall and I have minutely compared with *F. Americana*. We are unable to find the slightest difference between the Italian and the American flukes here named, and do not hesitate to make the unqualified statement that they are identical. The name *Distoma magnum* having the priority must stand as the specific name, and, as stated in my former note, No. 7, *carnosa*, *Americana*, and *Texanicum* must be regarded as synonyms.

This parasite occurs in *Antilope picta*, *Bos taurus*, *Cervus aristoteles*, *Cervus canadensis*, *Cervus dama*, *Cervus elaphus*, *Cariacus* (*Cervus*) *virginianus*.

Bureau of Animal Industry, U. S. Department of Agriculture, March 22, 1892.

Postscript.—Since writing the above, Dr. Francis has published a note (this Journal, July number) on this same species, in which he replies to my Note No. 7. Dr. Francis makes considerable stock out of the fact that I stated "this parasite has, however, been known for *some time* (italics are by Dr. Francis) to the Bureau of Animal Industry." He also quotes Dr. Dinwiddie's report of 1889, and mentions the fact that Dr. Curtice found the same flukes in Colorado. He also accuses me of not having the "fairness to mention the work of others." Were it not for this last sentence, I should not feel called upon to reply to the note of my colleague, Dr. Francis; but a scientist, especially one occupying a government position, cannot allow a charge of being dishonorable to pass without replying to it.

The present postscript is not for the purpose of decrying anybody's work, nor for the purpose of entering into a personal discussion with one or any of my American colleagues. It is entirely in a scientific and friendly spirit that I reply to Dr. Francis' note, in order to convince him and others who may possibly share his opinion, that he has misunderstood the object of my former Note (7), that he has misconstrued the statements, and finally, that he has been too hasty in charging me with a dishonorable act, a charge which I call upon him to withdraw as publicly as he made it, in case he cannot support it with evidence, or overthrow the statements of this postscript. In case anyone can overthrow these statements, I am perfectly willing to plead ignorance, or even carelessness, but under no circumstances will I accept Dr. Francis' charge of unfairness.

In the first place, let me state that I reported to Dr. Salmon for duty July 10th, 1891, and of course cannot be held responsible for any publication or statement in regard to parasites which any member of the Bureau made before that date.

Now let us see what I meant by the statement that this parasite "has been known to the Bureau for some time." There is a bottle in the Bureau collection containing specimens of *Distoma magnum*, labelled "*Distomum hepaticum, Kansas Cattle, Liver and Lungs, June, 1887, Colorado Springs.*" The name of the collector is not attached to the bottle, but I assume it was my friend and predecessor, Dr. Curtice, for he was in the West in 1886 and 1887, and mentions this parasite in an article in the American Veterinary Review, p. 390, 1887, under the name *D. hepaticum.* Dr. Curtice was a member of the Bureau Staff in 1887, hence "*This parasite has been known to the Bureau for some time.*" Dr. Curtice afterward came to the conclusion that this parasite was probably not *D. hepaticum* (personal conversation.)

Dr. Francis' interesting Bulletin bears the date of October, 1891. The first sentence of part II, on "*Distomum Texicanum,*" is: "It is now about three years since I saw this animal for the first time." The date of manuscript is not attached to his article, but it will be only fair to allow four or five months for the printer. This would bring us to May or June, 1888, as the first time he saw *D. magnum.*

We saw above that Curtice's specimens bear the label June, 1887; that is, Curtice's specimens antedate Francis' observation by one year. Curtice was in Colorado Springs in the spring of 1887, and probably collected these specimens at that time.

As for Dr. Dinwiddie's paper, that bears the official date, March 9, 1889, namely, nearly two years later than the time when this parasite became known to the Bureau.

*Why I did not mention these papers in my former Note* (7). The reason is very simple indeed: I was not writing an article on the distribution and occurrence of this parasite; I was simply making out the correct specific name of the animal, and hence mentioned only papers bearing upon that point. In making up the specific name of a species, zoologists are held strictly to the following rule, passed by the International Zoological Congress, Paris, 1889:

"LAW OF PRIORITY.

"35. The name attributed to each genus, or to each species, can only be that name which was first assigned to it, on the condition :

"*a*—That this name has been clearly and sufficiently described.

"*b*—That the author has applied the rules of binomial nomenclature."

(N. B.—Zoologists generally accept a figure or the original specimen as a substitute, in case there is any doubt in regard to the sufficiency of the original description.—C. W. S.)

In my Note No. 7, I held strictly to the above rule ; I implied that Hassall's name, "*carnosa*," could not be accepted because it was preoccupied, a fact already acknowledged by Hassal in print, and stated that his name, "*americana*," must be accepted in preference to Francis' name, "*Texanicum*," since the former antedated the latter. In this, *it was not a question as to who saw the parasite first ; it was simply a question as to who printed it first.* However, as I have since proven that "*americana*" is the same as Bassi's *D. magnum* (1875), the name "*americana*" also must be dropped and "*magnum*" accepted. It is interesting to note that Leuckart has examined both "*americana*" and "*magnum*," and comes to the same conclusions which I have stated above : i. e., that the two flukes are identical. Geheimrath Leuckart did not know that I had specimens of "*magnum*," nor did I know that he had specimens of that species ; so that our demonstration was made entirely independent of each other. Although we had exchanged letters upon the subject, expressing our belief that the two parasites were identical, neither of us knew of the other's ideas upon the subject when we first came to our conclusions. Although my manuscript is dated before Leuckart's article appeared in print, his article bears a printed date anterior to mine, so that his demonstration of the identity of *magnum* and *americana* takes precedence over mine. Even if there were any doubt in regard to this point, it would be almost impossible for me to imagine my having a discussion in regard to priority with Leuckart, a man of whom I have such a high opinion as a scientist, as my teacher and friend.

As for the charge which Dr. Francis makes against Dr. Hassall, the whole affair occurred while I was with Pasteur in Paris, before I was appointed to the Bureau. Hence it is a matter which does not concern me in the least, and one upon which I would have a right to express an opinion in print only at the request of the two gentlemen concerned. This does not in the slightest degree retract or contradict my statement at the end of Note 7, to the effect that "to Dr. Hassall is due the credit of insisting upon a specific distinction between *D. hepaticum* and the large liver fluke," etc. *I made that statement from printed articles, the only evidence admitted by scientists upon a question of this nature.*

Now one word more, and I will close. The subject of parasitism gives a very broad field for work ; there are but few men in America who are giving special attention to the subject of animal parasites, and there is enough material to keep us all busy. If, however, we expect to advance in this work, we must pull together and keep out of personal attacks upon the honor or fairness of our colleagues. If one of us makes a mistake, let another correct it, either in print or by personal letter, and let the author acknowledge the mistake with thanks rather than with personal enmity. Personal attacks upon a colleague's honor or fairness do not aid in advancing science, and should be made only in case of necessity.

I send a copy of this postscript to Dr Francis at the same time that I send the manuscript to the Journal, so that he may have an opportunity to reply in the same number in which this appears.

BUREAU OF ANIMAL INDUSTRY, U. S. DEPT. OF AGRICULTURE, July 19, 1892.

